# Shared trauma during the COVID‐19 pandemic: Psychological effects on Israeli mental health nurses

**DOI:** 10.1111/inm.12996

**Published:** 2022-03-28

**Authors:** Sagit Dahan, Galit Levi, Ronen Segev

**Affiliations:** ^1^ Lev‐Hasharon Mental Health Medical Center Netanya Israel; ^2^ 54619 Department of Nursing Sciences Ruppin Academic Center Emek‐Hefer Israel

**Keywords:** COVID‐19, posttraumatic growth, psychiatric nursing, psychological trauma, resilience

## Abstract

Mental health nurses, tasked with the constant care of clients undergoing mental health treatment, have faced unique challenges arising from the uncertain outcomes of the COVID‐19 pandemic. The shared exposure of both nurses and their patients to a traumatic event such this pandemic leads to additional challenges and ways of coping. The psychological effects of this shared trauma on mental health nurses arising from the pandemic are the subject of this study. An online survey was used to examine personal levels of anxiety and concern, personal and national resilience (NR), and posttraumatic growth (PTG) among 183 mental health nurses working in mental health services in Israel. Overall, the study revealed moderate levels of concern and relatively low levels of anxiety, with significant negative correlations between personal and NR and levels of concern and anxiety. Higher levels of personal and NR were related to lower levels of concern and anxiety, and there was a significant positive correlation between assessments of personal resilience and NR. A significant positive correlation was found between personal and NR and PTG. Higher religiosity was associated with higher resilience, and higher professional seniority was related to higher PTG. Finally, results for particular demographic subgroups indicate that in Israel, special attention should be given to those mental health nurses who have immigrated to Israel, are non‐Jews or have less professional experience.

## Introduction

The COVID‐19 pandemic has posed unique challenges to mental health nurses (Foye *et al*. 2021). Recent studies have demonstrated that the pandemic has characteristics consonant with a global traumatic event, with evidence from populations in Italy (Forte *et al*. 2020), China (Lai *et al*. 2020), and Israel (Lahav 2020). This global trauma is a collective one simultaneously affecting therapists and their patients (Masiero *et al*. 2020). Previous research has shown that while some who experience disasters, such as the COVID‐19 pandemic, will inevitably develop mental health problems following a disaster; others, will continue to function well and may even have positive emotional experiences resulting from the traumatic event (Brooks *et al*. 2020). This study aims to examine both the negative and positive psychological effects of the COVID‐19 crisis on mental health nurses.

## Background

Mental health nurses routinely face concrete stressors and professional challenges in their workplace (Foster *et al*. 2019). Their stress is a product of their inherently demanding vocation, which, under typical conditions, involves being exposed to verbal and physical violence, among other threats, and having to cope effectively with patients’ suicidal ideations (Foster *et al*. 2020). During the pandemic, mental health nurses found themselves dealing with a unique, shared traumatic reality. Shared traumatic reality refers to occurrences in which therapists and patients are simultaneously exposed to a collective trauma (Day *et al*. (2017). ‘Shared trauma is a phenomenon born out of a traumatic event, be it an individual or collective trauma, that is experienced at all levels – worldwide/multinational, societal, community, interpersonal, intrapsychic’ (Figley (2021) Foreword. In Tosone (2021) p ix). While this phenomenon has been extensively researched among social workers (Tosone 2021), studies of nurses are scarce.

Shared traumatic reality can cause damage, but it can also induce change, with studies showing that a shared experience of a traumatic reality can lead to both positive and negative outcomes (Baum 2014; Day *et al*. 2017; Nuttman‐Shwartz 2016; Tosone 2021). While nurses working in a shared traumatic reality may perceive their work as stressful and even traumatic, this reality may also spur posttraumatic growth (PTG) (Lev‐Wiesel *et al*. 2009), defined as ‘positive change that occurs as a result of the struggle with highly challenging life crises’ (Tedeschi & Calhoun 2004, p1).

A recent review of the positive aspects of trauma in response to the COVID‐19 pandemic argues that positive outcomes are also possible, as emphasized in the trauma literature on resilience, coping strategies, and PTG (Finstad *et al*. 2021). Resilience enables nurses to adapt positively to stressors and distress. A complex and dynamic process, resilience changes over time and context and involves both individual traits and external resources (Cooper *et al*. 2020). Resilience may be one of the central capacities required for developing effective therapeutic relationships, along with mental health nurses’ experience, knowledge, and clinical skills. The existing literature has focused primarily on individual mental health nurses’ resilience rather than environmental factors that may affect it. While mental health nurses’ resilience across cultures and the cultural and environmental factors that may influence understandings and expressions of their resilience is beyond the scope of this article, further research should be conducted on these issues (Foster *et al*. 2019).

National resilience (NR) is people's subjective perception of an entire country’s capacity to withstand crisis and recover as quickly as possible (Kimhi & Eshel, 2019). Recent studies (Ballada *et al*. 2021; Kimhi *et al*. 2020) proposed four elements that identify NR during the COVID‐19 crisis: individuals’ identification with their country, a sense of solidarity, a sense of social justice, and trust in public institutions. Previous studies have found a negative correlation between national and personal resilience and distress symptoms (Foster *et al*. 2019; Kimhi *et al*. 2020).

To the best of our knowledge, this study represents the first time the psychological effects of the COVID‐19 crisis on mental health nurses, who faced a shared traumatic reality and a stressful work environment, has been examined. We hypothesize that, as with other traumatic events, the COVID‐19 crisis has both negative and positive psychological effects on mental health nurses.

## Methods

In this study, negative psychological effects were assessed through analysing mental health nurses’ concerns and anxiety, and positive effects through analysing their personal resilience, NR, and PTG.

### Research design

A cross‐sectional study was carried out between April 1 and 30, 2020. STROBE reports for cross‐sectional studies (Vandenbroucke *et al*. 2007), were used in this study.

Participants: The research sample included 183 mental health nurses, all members of the Psychiatric Nursing Association in Israel. The participants worked at Israeli mental health centres, in psychiatric wards at general hospitals, and as community mental health nurses. Their ages ranged from 24 to 66 years old (M = 47.37, SD = 10.71) (Table 1).

**Table 1:** Characteristics of the sample of mental health nurses (*N* = 183)

Characteristic	N	%
Gender		
Male	64	35.0%
Female	119	65.0%
Country of origin		
Israel	102	55.7%
Former USSR	69	37.7%
America	7	3.8%
Europe	3	1.6%
Asia	1	0.5%
Other	1	0.5%
Family status		
Single	20	10.9%
Lives with a partner	4	2.2%
Married	129	70.5%
Divorced	25	13.7%
Widow	5	2.7%
Nurse education		
RN	69	55.7%
RN + clinical course	9	4.9%
Academic	16	8.7%
Academic + clinical course	87	47.5%
RN, MA	2	1.09%
Job description		
Staff Nurse	102	55.7%
Deputy Nurse or Head Nurse	39	21.3%
Nursing Director	42	22.9%
Religion		
Jewish	138	75.4%
Muslim	31	16.9%
Christian	6	3.3%
Druze	1	0.5%
Other	7	3.8%
Religiosity		
Secular	114	62.3%
Traditional	52	28.4%
Religious	12	6.6%
Other	5	2.7%
Professional seniority		
Up to 5 years	27	14.8%
6–10	14	7.7%
11–15	12	6.6%
16–20	24	13.1%
21–25	28	15.3%
26–30	36	19.7%
30+	42	23.0%

### Study setting

#### Data collection

An invitation to participate in an online survey was sent by text message to 800 registered members of the Israeli Psychiatric Nursing Association. A total of 183 participants (a response rate of 23%) provided valid, complete data, which are included in the analysis. The survey instruction included information on the purpose and significance of the study and required participating nurses to expressly consent to participate by clicking on an ‘Agree’ button before beginning the survey. Participation in the study was voluntary and anonymous. The study was approved by the IRB of Lev‐Hasharon Mental Health Medical Center (LH3/2020).

#### Analysis

To assess the negative effects of the COVID‐19 pandemic on mental health nurses, we probed their concerns and anxiety. Concern was assessed using questions that probed their concern about the virus for themselves, for relatives, and for the larger economic and political situation. Examples included: ‘How concerned are you about being infected by COVID‐19?’ and ‘How concerned are you for your ability to cope with the disease if you get it?’ (Cronbach’s α = 0.83). Eight questions were included, and answers were rated on a Likert scale of 1–5.

Respondents’ degree of anxiety was assessed with the seven‐item Generalized Anxiety Disorder Scale (GAD‐7), in which scores ≥10 indicate likely generalized anxiety disorder (Spitzer *et al*. 2006). In general, higher scores indicate higher anxiety levels. Scores were derived from the average response for all items (Cronbach’s α = 0.84).

To assess the positive psychological effects of the pandemic among mental health nurses, we examined personal resilience, NR, and PTG. We used an abridged version of the Connor–Davidson Resilience Scale (Campbell‐Sills & Stein 2007), a self‐report questionnaire of 10 items, using the Hebrew translation by Fridenzon (2011), to test for personal resilience. The questionnaire had convergent validity (Cronbach’s α = 0.88).

The National Resilience Questionnaire included 13 items on a scale ranging from 0 (very low) to 5 (very high). Examples of items include: ‘In a national crisis, the entire Israeli society will be behind the decisions of the government and its leader’ and ‘Israel is my home and I do not intend to leave it.’ The internal reliability of the scale was measured at Cronbach’s α = 0.90. (Kimhi *et al*. 2019). The measure of NR was computed by the average score for responses.

PTG was examined using the Questionnaire PTG‐Inventory. The Hebrew translation by Laufer and Solomon (2006) of the original scale by Tedeschi and Calhoun (1996) was used. This questionnaire, with 21 statements on the lifestyle and feelings of the examinee, evaluates positive changes reported by a respondent that occurred following exposure to a traumatic event. Participants were asked to respond to the following overall instructions: ‘For each of the following statements, state the extent to which this change has occurred in your life as a result of coping with the COVID‐19 pandemic,’ followed by a list of particular issues in the respondent’s life. Responses were scored on a 4‐point Likert scale from 1 (no change) to 4 (significant change). The questionnaire has structural validity, internal consistency (for the overall score and for each scale separately), and test–retest reliability (Cronbach’s α = 0.92). The measure of PTG was computed by the average of these items.

Data analyses were performed using SPSS Statistics 23 (IBM 2015). We examined the descriptive statistics of the research sample and the main research variables. To test the research hypotheses, we used Spearman correlation analysis, one‐way ANOVA analysis, and an independent sample *t*‐test. To predict anxiety, personal and NR, PTG, and the socio‐demographic variables of the sample, a linear hierarchical regression analysis was performed.

Significance was set to *P* < 0.05.

## Results

The results indicated that the level of concern for COVID‐19 was moderate (M = 3.20 ± 0.82), and the level of anxiety was relatively low (M = 1.50 ± 0.49). The level of personal resilience was high (M = 3.09 ± 0.61), and the level of NR was high (M = 3.44 ± 0.66). The level of PTG was moderate (M = 3.01 ± 0.81) (Table 2).

**Table 2:** Descriptive statistics of the main research variables

Variable	Scale	M	*SD*	Range
Concern	1–5	3.20	0.82	1.13–5.00
Anxiety	1–4	1.50	0.49	1.00–4.00
Personal resilience	0–4	3.09	0.61	1.10–4.00
National resilience	1–5	3.44	0.66	1.23–5.00
Posttraumatic growth	0–5	3.01	0.81	0.64–4.77

Significant negative correlations were found between personal resilience and levels of concern (*r_s_* = −0.17, *P* < 0.05) and anxiety (*r_s_* = −0.24, *P* < 0.01), with a higher level of personal resilience associated with lower levels of concern and anxiety (Table 3). In addition, significant negative correlations were found between NR and levels of concern (*r_s_* = −0.21, *P* < 0.01) and anxiety (*r_s_* = −0.14, *P* < 0.05). Finally, we found a significant positive correlation between personal and NR (*r_s_* = 0.25, *P* < 0.01).

**Table 3:** Relationship between personal and national resilience, levels of concern, and anxiety (*N* = 183)

	1	2	3	4
1. Personal resilience	–			
2. National resilience	0.25**	–		
3. Concern	−0.17*	−0.21**	–	
4. Anxiety	−0.24**	−0.14*	0.42**	–

*P < 0.05, **P < 0.01.

The results of a Spearman test on the relationships between personal and NR and PTG are given in Table 4. A significant positive correlation was revealed between personal resilience and PTG (*r_s_* = 0.24, *P* < 0.01). We also found a significant positive correlation between NR and PTG (*r_s_* = 0.29, *p* < 0.01).

**Table 4:** Relationship between personal and national resilience, and posttraumatic growth (*N* = 183)

	1	2	3
1. Personal resilience	–		
2. National resilience	0.25**	–	
3. Posttraumatic growth	0.24**	0.29**	–

**P < 0.01.

### Predictive model

A linear hierarchical regression analysis was performed to predict anxiety, personal and NR, PTG, and socio‐demographic variables (Table 5). Some socio‐demographic variables could significantly predict NR (*F* (8, 176) = 6.10, *P* < 0.01). The regression coefficients show that predictors of religion and religiosity had a significant positive contribution, adding 18% to the model variance. Being Jewish and having higher religiosity were related to higher NR. The regression for the prediction of PTG and socio‐demographic variables was significant (*F* (8, 176) = 3.61, *P* < 0.01). Religiosity and professional seniority had a significant positive contribution, adding 15% to the model variance. Higher religiosity levels and higher professional seniority were related to higher PTG (Table 6).

**Table 5:** Hierarchical regression for the prediction of national resilience and the socio‐demographic variables

Predictors	*B*	SE	*β*	*t*	*P*	*R* ^2^
Gender (1 = male)	−0.11	0.11	−0.07	−0.98	0.32	0.18
Age	0.01	0.01	0.15	1.17	0.24	
Birth country (Israel)	−0.12	0.10	−0.09	−1.19	0.23	
Religion (Jewish)	0.37	0.13	0.23	2.72**	0.01	
Religiosity	0.21	0.08	0.20	2.45*	0.02	
Professional seniority	0.04	0.04	0.11	0.89	0.37	
Administrative (1 = yes)	0.10	0.10	0.07	0.98	0.32	

*P < 0.05, **P < 0.01.

**Table 6:** Hierarchical regression for the prediction of posttraumatic growth and socio‐demographic variables

Predictors	*B*	SE	*β*	*t*	*P*	*R* ^2^
Gender (1 = male)	0.12	0.13	0.07	0.92	0.35	0.15
Age	−0.01	0.01	−0.07	−0.52	0.59	
Birth country (Israel)	0.02	0.12	0.02	0.19	0.84	
Religion (Jewish)	−0.09	0.16	−0.05	−0.58	0.56	
Religiosity	0.38	0.10	0.30	3.60**	0.00	
Professional seniority	0.10	0.05	0.26	2.04*	0.04	
Administrative (1 = yes)	0.06	0.14	0.04	0.51	0.60	

*P < 0.05, **P < 0.01.

### Differences in concern, anxiety, personal and NR, and PTG by country of origin

Differences in concern, anxiety, personal and NR and PTG between participants according to whether they were born in Israel or elsewhere were examined with an independent sample *t*‐test (Table 7). We found significant differences between participants according to their birthplace within or outside of Israel in PTG (*t* (181) = 2.44, *P* < 0.05). The level of PTG was significantly higher among participants who were born in Israel than among those born elsewhere. There were, however, no significant differences in concern, anxiety, or personal/NR related to the place of birth.

**Table 7:** Differences in concern, anxiety, personal and national resilience, and posttraumatic growth by country of origin (*N* = 183)

Measure	Born in another country *N* = 81		Born in Israel *N* = 102		*T*
	M	SD	M	SD	
Concern	3.27	0.80	3.10	0.83	1.39
Anxiety	1.50	0.53	1.50	0.44	0.03
Personal resilience	3.12	0.57	3.05	0.66	0.69
National resilience	3.38	0.70	3.52	0.61	−1.39
Posttraumatic growth	3.14	0.76	2.85	0.85	2.44*

*P < 0.05.

## Discussion

The COVID‐19 pandemic presents an unprecedented opportunity to study the impact on mental health nurses of experiencing a shared trauma together with their clients. Both feared for their own personal safety and well‐being, as well as for the health of those close to them. Like other nurses, mental health nurses had to navigate between their desire to fulfil the professional roles that define them and give them meaning, despite the unique stresses of the workplace, and their desire to ensure the health of their families at home (Wu *et al*. 2020). Mental health nurses, however, must manage both the pandemic’s stressors and the high degree of stress typical of their occupation. Building on other research (Baum. 2014), this study found that there are both negative and positive psychological effects on mental health nurses arising from such a shared traumatic reality.

In April 2020, in the middle of the first COVID‐19 wave in Israel, when public and health workers were expressing significant concerns about the growing morbidity and mortality associated with the pandemic, Israeli mental health nurses expressed moderate levels of concern, and their level of anxiety was low. Their levels of personal and NR were high, and their level of PTG was moderate.

Our findings differ from those of another study conducted in Israel at the same time. In a sample of 503 Israeli citizens drawn from the general population, Shapiro *et al*. (2020) found that almost a quarter expressed high or very high levels of anxiety or worry. Their study, however, did not focus on healthcare workers. Reporting findings similar to ours, Kameg *et al*. (2021) focused on mental health nurses during the pandemic and found only mild rates of anxiety, and de Pinho *et al*. (2021) comparing mental health to non‐mental health nurses found that mental health nurses experienced less depression, anxiety, and stress and used more strategies to promote mental health during the pandemic than did other nurses. Collectively, these studies indicate that mental health nurses successfully manage significant stressors. To the extent that their success relies on their ability to deploy familiar self‐care strategies to manage both workplace and society‐wide stress, these findings reveal the importance of regular self‐care professional training and personal development, especially as the foundation for work with clients in shared trauma reality (Day *et al*. 2017). Another factor that may explain the lower levels of concern and anxiety expressed by mental health nurses in response to the pandemic may be their higher levels of personal resilience – a hypothesis supported by the negative correlation we found between measures of resilience and levels of concern and anxiety. Similarly, Kimhi *et al*. (2020) find that resilience is a good indicator of people’s ability to cope with various crises and threats.

In the COVID‐19 context, the link between personal resilience, NR, and PTG have not been deeply studied. This study revealed that personal resilience, NR, and PTG were positively related to one another.

Recent studies show that personal resilience and PTG were positively correlated among nursing students and healthcare workers during the current COVID‐19 pandemic (Kalaitzaki & Rovithis, 2021; Yildiz 2021) However, Itzhaki *et al*. (2015) did not find a correlation between resilience and PTG among mental health nurses who were exposed to violence. Adaptive coping strategies and resilience contribute to the development of PTG (Finstad *et al*. 2021). Apparently, this phenomenon explains our findings.

NR has not been examined among mental health nurses. However, our findings are comparable to those of Kimhi *et al*. (2020), who found a negative correlation between NR and distress symptoms during the COVID‐19 crisis in the Israeli general population (Kimhi *et al*. 2020). In Israel, during the COVID‐19 pandemic, while the unemployment rate was very high, healthcare workers were nevertheless strongly supported by the government and no nurses lost their jobs. Additionally, government‐provided special structures for children of healthcare workers, so they would not be concerned about closed schools and daycare. Higher NR related to lower distress symptoms could be explained by this feeling of support and security, although a causal connection cannot be established. Further research is needed to support this explanation.

Our study found a significant positive correlation between personal resilience and NR, with higher levels of personal resilience being related to higher levels of NR. This finding is consistent with earlier studies indicating that people who have a greater capacity to bounce back from adversity are more likely to trust their national leaders and government institutions to resolve crises and maintain stability in the country (Callueng *et al*. 2020; Kimhi & Eshel, 2009, 2019).

To the best of our knowledge, the current study is the first to show a positive connection between NR and PTG following COVID‐19. Previous research argues that NR is the best predictor of posttraumatic recovery, in times of war (Kimhi & Eshel 2009). A review of the literature indicates a rather small number of empirical investigations of NR and its association with antecedent variables (Kimhi & Eshel 2019).

This study’s finding of a moderate level of PTG among mental health nurses is consistent with the findings of several studies on nurses in a shared traumatic wartime reality (Lev‐Wiesel *et al*. 2009), mental health nurses with exposure to violence (Ithaki *et al*. 2015), and frontline nurses during the COVID‐19 pandemic (Chen *et al*. 2021; Pan Cui *et al*. 2021). Their role of being helpers, responsible for others, and needed by their clients at times of crisis, in addition to being acknowledged as an essential profession by the authorities and public, all served as sources of growth (Lev‐Wiesel *et al*. 2009). Our results indicate that experiencing positive psychological change can coexist with a unique emergency like the COVID‐19 pandemic.

In addition, our study found that the PTG of mental health nurses is generally affected by religiosity and professional seniority. Although PTG among mental health nurses is seldom examined, our findings are comparable to earlier studies indicating that religious and spiritual connection are particularly strongly related to PTG (Lehmann & Steele 2020; Shaw *et al*. 2005), as well as those of a recent study conducted among frontline nurses fighting COVID‐19 that showed relationships between professional seniority and PTG (Pan Cui *et al*. 2021). This could be attributed to additional years of abundant nursing and life experience, and of greater self‐confidence and appreciation of life (Ogińska‐Bulik *et al*. 2021). Lehmann and Steele (2020) highlighted the social support function of religious participation. These findings suggest that disaster survivors who identify as religious may tend to draw on their religion/spirituality to cope with disaster‐related adversity, such as during the recent pandemic.

Finally, significantly greater PTG was reported among participants born in Israel than among those born in another country. This aligns with other recent studies where immigrants presented lower mental health than non‐migrants in times of COVID (Solà‐Sales *et al*. 2021) and was more likely to both report anxiety and to seek professional mental health services than were native‐born Israelis during the COVID‐19 pandemic (Shapiro *et al*. 2020). Alternatively, the lower level of PTG among immigrant mental health nurses may be influenced by their immigration, which involves significant losses in a number of areas: knowledge of their physical and cultural environment, status, economic and social resources, language and identity, as well as a sense of community. Immigrants may lose their sense of identity and their role in the world. As a result, the immigration experience is very stressful, even traumatic. Professionals need to develop new ways of thinking about how to work with these populations, not only to reduce stress but also to promote personal growth (Tsong *et al*. 2021).

### Limitations

Data collection occurred at the height of the first wave when the subjects experienced the peak of their work pressure and the uncertainty about the nature of COVID‐19. As a result, their willingness and ability to respond to a survey were likely relatively limited. Additionally, those who did respond may have been either less pressured at work or the more resilient nurses. The study relatively small sample size, surveying a single time point in a single country, should also be taken into account when interpreting the results, which limited the ability of findings' generalization.

## Conclusions

The COVID‐19 pandemic has added new challenges to the already stressful workplace, relational dynamics, and mechanisms of coping of mental health nurses. Little research has been published on this issue, and it would be useful for future research to focus on these nurses’ experiences and how they are affected by a shared traumatic reality.

Overall, this study describes the psychological effects of the COVID‐19 pandemic among mental health nurses. These results highlight the importance of assessing psychological effects among mental health nurses, who are providing psychological assistance to clients who are themselves under severe psychological stress, intensified by the pandemic.

Mental health nurses, like other healthcare workers, need mental support to enable them to care for their patients. To relate to our study, higher personal and NR suggest that mental health nurses may have felt more supported. To provide positive and constructive working conditions when under extreme stress, such as during the current pandemic, hospital and ward managers should encourage staff, support them, and be attentive to their concerns and needs, particularly those who are immigrants or non‐Jews, and those with little professional experience.

## Relevance for Clinical Practice

This study highlights critical factors in the work of mental health nurses during the major traumatic event of the COVID‐19 pandemic. It indicates that mental health nurses with higher personal and NR, lower anxiety, and concerns, managed their shared traumatic experience of COVID‐19 with higher levels of PTG.

Suggesting that organizations could support nurses during a traumatic event such as the COVID‐19 pandemic and the government could establish and implement more effective workplace policies that may contribute to the development and implementation of more effective responses to traumatic events for mental health nurses and their clients.

## Funding

This study was conducted without any outside funding.

## Ethics Approval

The study was approved by the IRB of Lev‐Hasharon Mental Health Medical Center (LH3/2020).
